# Review of systemic and syndromic complications of cannabis use: A review

**DOI:** 10.1097/MD.0000000000032111

**Published:** 2022-12-09

**Authors:** Jay Shah, Olga Fermo

**Affiliations:** a University of Queensland/Ochsner Clinical School, Brisbane, QLD, Australia; b Department of Neurology, Mayo Clinic Florida, Jacksonville, FL, USA.

**Keywords:** adverse effect, cannabis, chronic pain, complications, marijuana

## Abstract

Purpose of review: Prescribed and non-prescribed cannabis use is common. Providers in specialties treating chronic pain – primary care, pain management, and neurology–will be coming across medical cannabis as a treatment for chronic pain, regardless of whether they are prescribers. It is important to be aware of the systemic and syndromic complications of acute and chronic cannabis use in the differential diagnosis of cardiac, cardiovascular, cerebrovascular, gastrointestinal, and psychiatric disorders.

Recent Findings: Medical cannabis is legal in 36 states. Studies have shown several potentially serious adverse effects associated with cannabis use.

Summary: Cannabis use has the potential to cause several complications that can be easily overlooked without a preexisting high index of suspicion.

## 1. Introduction

The use of marijuana (or cannabis) for medicinal and therapeutic purposes has been debated for hundreds of years. A European physician published his discovery of the muscle-relaxant, anticonvulsant, and analgesic properties of medical marijuana in 1839; since then, its use has continued to grow.^[1]^ Over time, a wide variety of indications have been associated with medical marijuana use (with varying literature support); however, the most common indication for prescription is chronic pain. In the United States, approximately 28% of patients are diagnosed with either chronic pain or high-impact chronic pain, according to the National Center for Health Statistics data brief. High-impact chronic pain is defined as pain lasting more than 3 months, with restriction in at least 1 major activity. Globally, the 12-month prevalence of chronic pain is estimated to be 37%.^[2]^ Cannabis modulates the central nociceptive pathways via the cannabinoid receptor type 1 (CB1) system, providing a treatment strategy for chronic pain.^[3,4]^

There has been a significant increase in marijuana use in the United States over the past 20 years, with a large proportion of marijuana use being illicit. A 2006 500-patient study evaluating comprehensive pain management treatment showed that 11% of patients had a positive urine drug test result for cannabinoids.^[5]^ In the past 5 years, however, there have been significant changes in the legality of marijuana use in the United States, all of which contribute to our current pain management environment. As of April 2021, recreational use of cannabis is legal in 17 states, medical use of cannabis (i.e., legal use only permitted via medical permission) is legal in 36 states, and 13 states have decriminalized its use.^[6]^ Recent CDC estimates show that 22.1 million people use marijuana across the United States each month, with the risk of addiction ranging from 1 in 10 users for individuals who start using marijuana after the age of 18, to 1 in 6 users for individuals who started using marijuana before the age of 18. Table 1 shows the current list of states and territories and their updated positions on decriminalization and legalization. As political climates continue to shift and more states legalize and decriminalize marijuana use, physicians treating chronic pain have found that an increasing proportion of their patients have concomitant authorized marijuana use.

**Table 1:** United States’ current legal standpoint on marijuana/ cannabis.^[6]^

State	Legality	State Regulated Cannabis Programs	Decriminalized
Alabama	Mixed	Yes	No
Alaska	Legal	Yes	Yes
Arizona	Legal	Yes	Yes
Arkansas	Mixed	Yes	No
California	Legal	Yes	Yes
Connecticut	Mixed	Yes	Yes
Delaware	Mixed	Yes	Yes
District of Columbia	Legal	Yes	Yes
Florida	Mixed	Yes	No
Georgia	Mixed	CBD/Low THC only	No
Hawaii	Mixed	Yes	Yes
Idaho	Illegal	No	No
Illinois	Legal	Yes	Yes
Indiana	Mixed	CBD/Low THC only	No
Iowa	Mixed	CBD/Low THC only	No
Kansas	Illegal	No	No
Kentucky	Mixed	CBD/Low THC only	No
Louisiana	Mixed	Yes	No
Maine	Legal	Yes	Yes
Maryland	Mixed	Yes	Yes
Massachusetts	Legal	Yes	Yes
Michigan	Legal	Yes	Yes
Minnesota	Mixed	Yes	Yes
Mississippi	Mixed	Yes	Yes
Missouri	Mixed	Yes	Yes
Montana	Legal	Yes	Yes
Nebraska	Mixed	No	Yes
Nevada	Legal	Yes	Yes
New Hampshire	Mixed	Yes	Yes
New Jersey	Legal	Yes	Yes
New Mexico	Mixed	Yes	Yes
New York	Legal	Yes	Yes
North Carolina	Mixed	CBD/Low THC only	Yes
North Dakota	Mixed	Yes	Yes
Ohio	Mixed	Yes	Yes
Oklahoma	Mixed	Yes	No
Oregon	Legal	Yes	Yes
Pennsylvania	Mixed	Yes	No
Rhode Island	Mixed	Yes	Yes
South Carolina	Illegal	CBD/Low THC only	No
South Dakota	Legal	Yes	Yes
Tennessee	Illegal	CBD/Low THC only	No
Texas	Mixed	CBD/Low THC only	No
Utah	Mixed	Yes	No
Vermont	Legal	Yes	Yes
Virginia	Legal	Yes	Yes
Washington	Legal	Yes	Yes
West Virginia	Mixed	Yes	No
Wisconsin	Mixed	CBD/Low THC only	No
Wyoming	Illegal	CBD/Low THC only	No

CBD = cannabidiol, THC = tetrahydrocannabinol.

A large proportion of the literature on the positive effects of cannabis is based on observational studies. The most common medical indications for cannabis are chronic pain, neuropathic pain, epilepsy, spasticity, and chemotherapy-induced nausea and vomiting. Owing to the complex biopsychosocial nature of chronic pain and its management, many patients who visit the pain management clinic use cannabis as an adjunct to their regular regimen, and the potential synergistic effects of regular pain management protocols with cannabis use may mask the true impact of cannabis itself. A 2017 report released by the National Academy of Science, Engineering, and Mathematics evaluated randomized clinical trials of cannabis use in patients with neuropathic pain, complex regional pain syndrome, musculoskeletal pain, spinal cord injury, human immunodeficiency virus (HIV), and rheumatic pain.^[7]^ The study found an approximately 40% reduction in chronic pain-related symptoms. Another survey of Colorado citizens over the age of 60 years found that 54% of marijuana users were taking it for both recreational and medicinal reasons, primarily due to arthritis, chronic back pain, anxiety, and depression.^[8]^

As the use of medical marijuana increases in the United States, it is important for providers treating chronic pain to understand the full spectrum of risks and benefits associated with cannabis use (and abuse). The purpose of highlighting these risks is twofold. First, it is important to consider these risks when screening patients for baseline comorbidities when starting or recommending a formal medical cannabis programme. Second, we need to be mindful of the rare but negative consequences of cannabis use, as these could easily be overlooked without high clinical suspicion when patients present for the evaluation of new non-pain-related symptoms. This review aims to educate healthcare providers on the potential risks of long-term medical marijuana use, including several specific syndromic associations and drug interactions.

### 1.1. General adverse effects of medical marijuana

#### 1.1.1. Cardiac, cardiovascular, and cerebrovascular.

A well-known acute effect of marijuana consumption is an increase in the heart rate and blood pressure for 2 to 3 hours after ingestion. One study found a 20% to 100% increase in heart rate and supine blood pressure after use, an effect hypothesized to be driven by cannabis-induced vasodilation and subsequent reflex tachycardia.^[9,10]^ Arrhythmogenic effects have also been described - a systematic review of 6 cases showed an association between atrial fibrillation and smoking marijuana. This was hypothesized to be due to adrenergic stimulation, which shortens the action potentials and increases cardiac automaticity and micro-reentry. Similarly, catecholamine release associated with cannabis use has been shown to leads to ventricular arrhythmia.^[9]^

Another important consideration is the prothrombotic effect of cannabis use. One proposed mechanism is the impact of CB1 and cannabinoid receptor type 2 on platelet expression of glycoprotein IIb-IIIa and P-selectin, which, when coupled with endothelial damage and increases in heart rate and sympathomimetic activity, may precipitate myocardial and peripheral vascular events.^[11]^ There is evidence that cannabis has the potential to trigger myocardial infarction (MI). While the mechanism is unknown, hypotheses range from vasospasm to thrombotic plaque rupture, autonomic dysfunction, and abnormal platelet activation. The evidence for increased risk of MI is strongest in the acute setting, as a case-crossover study showed a nearly 5-fold elevated risk for MI in the hour after smoking marijuana, but no significantly increased risk after the first hour of use. (10) Cerebrovascular events have also been reported. A 2016 study found an association with cannabis use in patients aged 18 to 54 years old, as users had a 17% increased risk of hospitalization due to acute ischemic stroke.^[12]^ The evidence seems to point towards a more temporal relationship, similar to that seen with acute myocardial infarction. A systematic review of case reports on cannabis use and stroke found that 22% of patients experienced a repeat stroke after another exposure to cannabis.^[13]^

However, this risk is not limited to thrombotic events. Some case series have shown an association between cannabis use and recurrent myopericarditis and stress cardiomyopathy.^[14,15]^ While there are multiple confounders for all of the aforementioned conditions (e.g., concomitant tobacco usage, increased appetite and poor diet, poor health maintenance), it is important to at least consider patient risk factors for these conditions when determining whether to initiate treatment and to screen for recent marijuana use when evaluating cardiac, cardiovascular, and cerebrovascular concerns.

#### 1.1.2. Pulmonary.

Marijuana can pose a pulmonary risk with chronic use. Specifically, patients are at high risk of chronic inflammatory changes and subsequent obstructive pulmonary disease. While the literature is not as well established as for tobacco smoking and there is a risk of confounding, a study that evaluated long-term pulmonary effects when adjusted for smoking also found an increased risk of lung adenocarcinoma in cannabis users.^[16]^

### 1.2. Psychosocial

Psychiatric and substance use disorders have also been affected by long-term cannabis use, especially in patients with chronic pain. One major psychiatric consideration is the association between marijuana use and acute-onset catatonia. The latest DSM-V criteria for catatonia involve a range of manifestations, from psychomotor agitation to rigidity, emotional excitability, and absolute flat affect.^[17]^ The estimated prevalence of catatonia ranges from 7.8% to 36%, primarily in patients with preexisting psychiatric or medical condition.^[18]^ However, a review of catatonia episodes in patients with consistent cannabis use but no preexisting conditions showed that it served as a predictive value in the development of subsequent schizophrenia.^[19]^ While the link between schizophrenia and cannabis use is well described and taught, recognizing early signs that a psychosis-driven disorder may be incoming would be important in the management of patients with chronic cannabis use. Acute catatonia is often managed with benzodiazepines, such as lorazepam, as well as ECT in severe, refractory cases.^[19]^

There is an important association between cannabis use and the rate of opioid misuse in patients with chronic pain. While current guidelines do not recommend the use of opioids for nonmalignant pain, and opioid prescription rates have decreased with increased regulation, the potential for misuse of either drug is an important consideration. A prospective study conducted in 2006 showed a 21% increase in the risk of opioid misuse in patients with a positive cannabis drug test.^[20]^

Patients are at risk of psychiatric manifestations due to long-term cannabis use. Anxiety and depression often coexist with cannabis use and this relationship may be bidirectional. On the 1 hand, over 50% of patients with chronic pain report anxiety and depression, and surveys show that these are the 2 major reasons for use.^[21]^ On the other hand, marijuana use has been linked to the exacerbation of psychiatric illness, including schizophrenia, worsening anxiety, paranoia, and acute psychosis. Concomitant depression is also often seen with substance abuse disorder, and the literature is unclear as to which comes first.^[22]^ This feeds into the biopsychosocial model that we use to evaluate chronic pain and emphasizes the need to assess a psychiatric baseline of patients for which medical marijuana is being considered. It is also important to consider marijuana use as a potential cause of new-onset psychiatric concerns or worsening of existing mood disorders when evaluating whether therapy is appropriate.

### 1.3. Syndromic effects of marijuana

#### 1.3.1. Cannabinoid hyperemesis syndrome (CHS).

CHS is a functional gastrointestinal disorder that belongs to the same family as chronic nausea, vomiting, and cyclical vomiting syndromes. The disorder is associated with classic cyclical vomiting episodes associated with regular (often daily) cannabis use. Other notable aspects of the patient’s presentation include abdominal pain, which starts in the epigastrium and radiates diffusely.^[23]^ The mechanism of CHS is unclear. Chronic use and the long half-life of tetrahydrocannabinol (THC), as it is fat-soluble, increases the risk of repeated intoxication in settings of fasting and lipolysis. This is supported by animal models that showed antiemetic effects of low-dose THC, but proemetic effects at higher doses.^[24,25]^

Patients with CHS undergo 3 phases of the disease in their clinical course: prodrome, hyperemesis, and recovery. Prodrome is associated with temporal nausea, vomiting, and abdominal discomfort that can last for any number of months or years. Subsequently, patients often have repeated acute care visits during the hyperemetic phase with abdominal pain, high-volume vomiting, and intractable cyclical nausea.^[26]^ Patients often experience sympathetic overdrive during this phase as well, with tachycardia, hot flashes, and hypertension. Additionally, patients take hot showers to try to relieve their symptoms during this phase. A proposed mechanism is that cannabis may raise the core body temperature while decreasing skin temperature, which causes the patient to compensate by taking hot showers (to increase skin blood flow and heat dissipation).^[27]^ The diagnosis is often missed at first presentation due to a lack of awareness of the disorder.^[28]^ Potential complications of CHS are the same as those of other patients with intractable vomiting, acute kidney injury, electrolyte imbalances, esophageal injury, and the risk of esophageal rupture. Acute management of CHS includes volume resuscitation and the use of antiemetic medications, including ondansetron, benzodiazepines, haloperidol, and topical capsaicin.^[26]^ Capsaicin is an unexpected but interesting therapeutic intervention, as it increases skin heat sensation and subsequently reduces the sensations of nausea, vomiting, and discomfort. Capsaicin modulates the TRPV-1 receptor, which is found in proximity to the CB-1 and CB-2 receptors in the hypothalamus, gastrointestinal tract, and peripheral nervous system.^[26]^ Long term management of CHS utilizes a psychosocial and medication-based approach. Patients often start motivational or behavioral therapy for cannabis cessation, and medications such as gabapentin or N-acetylcysteine are used as adjuncts.^[29,30]^ Another consideration is the risk of cannabis withdrawal. Common symptoms include anxiety, insomnia, irritability, tension, and loss of appetite. Some studies have shown that tricyclic antidepressants, such as amitriptyline, are effective for managing withdrawal symptoms, but these medications have their own side effect profile (e.g., anticholinergic toxicity). Newer medications, such as dronabinol, are currently being evaluated for the management of cannabis withdrawal symptoms as they provide a controlled dose of THC (which may not often be true of non-medically acquired marijuana).^[31]^ Management of concomitant mood disorders, such as anxiety, has also been shown to reduce healthcare utilization and the frequency of vomiting episodes in CHS.^[32]^

#### 1.3.2. Reversible cerebral vasoconstriction syndrome.

Reversible cerebral vasoconstriction syndrome (RCVS) is a neurological complication of cannabis use that can potentially cause life-threatening cerebrovascular consequences. The hallmark of this disease is recurrent thunderclap headache with or without focal neurological deficits, including seizures caused by multifocal and reversible vasoconstriction, often known as multifocal intracranial stenosis (MIS).^[33]^ Multiple studies have attempted to link RCVS with cannabis use, with varying results. A systematic review of 62 cases in 2007 showed that 30% of the patients with RCVS had significant cannabis use.^[34]^ In 2011, another study examining patients with stroke and positive and negative cannabis screens found that 77% of the patients with positive cannabis screens had symptoms and imaging findings consistent with RCVS and MIS, and only 3% of the patients with negative cannabis screens had RCVS or MIS findings.^[35]^ Symptoms of RCVS follow a self-remitting course that lasts up to 12 weeks; however, pharmacological management with oral calcium channel blockers (nimodipine and verapamil) is considered for severe cases.^[36]^ Nimodipine is especially effective because of its ability to readily cross the blood-brain barrier, and is well known for its use in preventing vasospasm after subarachnoid hemorrhage. The early initiation of nimodipine is associated with a shorter clinical course and rapid remission of headache symptoms. Importantly, while glucocorticoids have been used in the past, recent data show that they worsen clinical symptoms and lead to the development of new ischemic lesions.^[37]^ Studies evaluating the short- and long-term prognosis of RCVS after discharge note that 80% of patients experience full functional outcome, while 20% experience some sort of deterioration prior to resolution. However, permanent functional loss was only observed in 10% of the cases.^[37]^ A 45-patient study evaluating long-term follow-up in patients with RCVS showed that more than half of the patients had a persistent headache, different from the thunderclap nature of RCVS, which caused some degree of functional discomfort.^[38]^

### 1.4. Medication interactions

As physicians continue to move towards making prescription decisions for medical marijuana, an important additional consideration will be interactions between cannabis-containing compounds and traditional pharmaceutical medications. The mechanism of action of cannabis in the modulation of is theorized to involve the CB1 pathway, which modulates CNS opioid signaling, and the cannabinoid receptor type 2 pathway, which modulates peripheral nervous system signaling. However, these are not the only receptors that cannabis-containing compounds, specifically THC, are hypothesized to possess.^[3,4]^

Systematic reviews evaluating in vitro and human studies on drug interactions have shown that THC compounds are potent modulators of the cytochrome P450 system, which is key for hepatic drug activation and metabolism. For example, THC and CBD are inhibitors of cytochrome P3A4 (CYP3A4) and cytochrome P2C9 (CYP2C9), and inducers of cytochrome P1A2 (CYP1A2).^[39]^ Bidirectional effects are seen with membrane-bound transporter systems as well, including multidrug resistance proteins (MDR) and glycoprotein P.^[40]^ What this means From a clinical standpoint, caution should be exercised with medications that are known to be metabolized or transported via 1 of these pathways, as the potency and efficacy of other prescribed medications may be altered by the introduction of cannabis. For example, warfarin is metabolized via the hepatic cytochrome P450 system, and the introduction of cannabis may prolong the effect of the anticoagulant and increase INR. Sildenafil has also been shown to have deleterious myocardial effects when combined with cannabis and lithium has been shown to have a longer half-life with cannabis in the system as well.^[41]^ Furthermore, a case series of tricyclic antidepressants interacting with cannabis showed an increase in anticholinergic activity in patients with both delirium and tachycardia.^[41,42]^ Table 2 shows select medications that use the Cytochrome and Glycoprotein pathways which are modulated by cannabis use.^[43]^ This is especially important in the context of polypharmacy. A thorough medication reconciliation would assist in ensuring the appropriate dosing of interacting medications, as well as THC content in prescribed medical marijuana. It is also important to be aware of these interactions when evaluating new or worsening side effects of previously tolerated medications after medical marijuana has been started.

**Table 2:** Potential cannabis modulations and medicine interactions.^[42,43]^

*Modulation*	*Inducer vs* Inhibitor	*Effect*	*Medications*
CYP3A4/ CYP2C9	Inducers	Increased THC breakdown = decreased availability	Antiepileptics
			Rifampin
			SGLT-2 inhibitors
CYP3A4/ CYP2C9	Inhibitors	Decreased THC breakdown = increased availability	Amiodarone
			Clopidogrel
			Fluorouracil
			Ketoconazole
			Protease inhibitors
			Tamoxifen
P-glycoprotein	N/A – P glycoprotein helps with modulation of toxin and drug efflux from the cell (as part of the drug ADME pathway)	Reduced P-glycoprotein expression in prolonged cannabis use	

THC = tetrahydrocannabinol, SGLT-2 = sodium-glucose co-transporter 2, ADME = absorption, distribution, metabolism, excretion.

## 2. Conclusion and next steps

With the growing annual prevalence of both recreational and medical cannabis use, the role of pain management physicians has expanded to increase patient management and monitoring. Important aspects of incorporating medical marijuana within practice include appropriate medication reconciliation, patient education prior to and during trial of use, and fellow-provider education on best practices in patient management. Future studies should be conducted to identify and trend the true incidence and prevalence of cannabis-associated adverse effects, and chronic pain management practice should be dynamic in its efforts to safely incorporate this therapeutic option into patient care.

## Author contributions

**Conceptualization:** Olga Fermo.

**Investigation:** Jay Shah.

**Supervision:** Olga Fermo.

**Writing – original draft:** Jay Shah.

**Writing – review & editing:** Olga Fermo.

## Correction

Jay Shah’s degree has been corrected to BS.
